# Effect of Polyphenols Extracted from *Rosa roxburghii* Tartt Pomace with Different Particle Sizes on Quality and Biological Activity of Noodles: A View of Molecular Interaction

**DOI:** 10.3390/foods14213679

**Published:** 2025-10-28

**Authors:** Keying Lin, Junjie Huang, Jichun Zhao, Xiaojuan Lei, Jian Ming, Fuhua Li

**Affiliations:** 1 College of Food Science, Southwest University, Chongqing 400715, China; lky203146@gmail.com (K.L.); mingjian1972@163.com (J.M.); 2 Westa College, Southwest University, Chongqing 400715, China; 3 Research Center for Fruits and Vegetables Logistics Preservation and Nutritional Quality Control, Southwest University, Chongqing 400715, China; 4 Chongqing Key Laboratory of Speciality Food Co-Built by Sichuan and Chongqing, Chongqing 400715, China

**Keywords:** texture, cooking properties, starch digestibility, antioxidant activity, quadratic regression models

## Abstract

The retention of polyphenols in thermally processed noodles is constrained by interactions with starch and glutenin, critically impacting functional properties (antioxidant activity, starch digestibility modulation) and quality attributes. Current understanding lacks quantitative links between initial pomace particle size, polyphenol behavior throughout processing, and the resulting noodle properties. This study systematically investigated how *Rosa roxburghii* pomace particle size (0.1–250 μm), fractionated into five ranges, governs polyphenol extractability, retention in fresh/boiled noodles, and their functional and quality outcomes. Mathematical modeling established quantitative particle size–property relationships. The results indicated that polyphenol release was maximized at the 1–10 μm particle size. Total phenolic retention in boiled noodles was highest with 0.1–1 μm pomace, while the retention of specific phenolics peaked with 60–80 μm pomace. Fresh noodle hardness and gumminess decreased significantly, particularly with extracts from 1 to 40 μm pomace, whereas boiled noodles showed increased chewiness/adhesiveness. All polyphenol-enriched noodles exhibited suppressed starch digestibility and enhanced antioxidant capacity. Robust quadratic regression models predicted key properties based on particle size. Molecular interactions (hydrogen bonding, hydrophobic contacts, π–cation stacking, salt bridges) between key phenolics (EGCG, hydroxybenzoic acid, gallic acid, quercetin, and isoquercitrin) and the gluten–starch matrix, critically involving residues Arg-86 and Arg-649, were identified as the underlying mechanism. These results demonstrate that precise control of pomace particle size regulates extract composition and molecular binding dynamics, providing a strategic approach to optimize functional noodle design.

## 1. Introduction

*Rosa roxburghii* Tratt has been valued historically as both a functional food and traditional medicine due to its numerous pharmacological effects and significant healthcare applications [1]. The health benefits of its fruit have been closely linked to its rich content of bioactive compounds, among which phenolic compounds have attracted substantial research interest [2,3,4,5]. Beyond direct health benefits, the utilization of *R. roxburghii* by-products aligns with sustainable food processing by transforming agricultural waste into value-added functional ingredients.

A major by-product of *R. roxburghii* fruit processing is pomace, which holds significant potential as it retains nearly 50% of the fruit’s phenolic compounds [2,6]. Critically, these phenolics primarily exist in bound forms within the pomace, which exhibit superior antioxidant activity compared to their free counterparts [2]. However, their covalent attachment to structural plant cell wall components severely limits their release during digestion, hindering bioavailability [7,8,9]. Unlocking this bound phenolic pool is therefore both a nutritional and industrial priority with direct implications for functional food development and circular bioeconomy strategies.

Micron- and nanoscale technologies offer a promising solution by reducing particle size, thereby disrupting the internal cohesion of the plant matrix and facilitating the liberation of bound phenolics [10]. Particle size reduction has been correlated with enhanced functionality, improved dough and baking properties [11,12,13], and increased bioaccessibility of antioxidant phytochemicals [14,15]. Consequently, these technologies provide an efficient means to release bound phenolics from pomace, broadening its application potential in the food industry.

Incorporating phenolic compounds into flour-based products can improve their functional activities and modify key starch characteristics, including crystalline structure, gelatinization properties, and retrogradation behavior, resulting in reduced starch digestibility [16,17]. Phenolics also influenced the physicochemical and sensory properties of enriched foods [18,19]. Thus, utilizing micron/nanoscale technologies to release pomace phenolics for incorporation into flour products represents a strategic approach to enhance polyphenol utilization efficiency while improving product quality and functionality. Despite these advantages, a critical challenge exists. Reducing particle size increases surface area, potentially enhancing solvent penetration and converting bound phenolics into bioaccessible free forms [20,21]. However, intensive milling can generate localized heat that can degrade heat-sensitive polyphenols [22]. This creates a critical “release vs. degradation” balance. Identifying the optimal particle size for net polyphenol release (maximizing liberation, minimizing degradation) and understanding release kinetics across the sub-micron to micron scale remain key challenges.

The retention of added polyphenols in cooked noodles after thermal processing (e.g., boiling) is constrained by processing conditions and interactions with key noodle constituents, particularly texture-governing starch and structural glutenin. Crucially, these retained polyphenols determine the noodles’ functional properties (antioxidant activity, starch digestion modulation) and quality attributes (texture, color, cooking properties) [23,24,25]. However, current studies lack a comprehensive understanding of two critical aspects: (1) how retained polyphenols interact with starch and protein to influence noodles’ functional properties and quality attributes and (2) how the initial pomace particle size quantitatively governs the entire pathway—from extraction efficiency to the final properties of cooked noodles (including texture, color, cooking loss, polyphenol retention/composition, starch digestibility, and antioxidant activity). Furthermore, neither quantitative modeling of particle size–property relationships in noodles nor explicit molecular docking analysis of polyphenol–starch–glutenin interactions has been conducted.

Polyphenols have been widely reported to enhance the functional properties of flour-based products [16,17,18,19]. Their effectiveness, however, depends largely on retention during processing and digestion, which is governed by both external processing conditions and internal interactions with starch and proteins [23,24,25]. Such an impact is further shaped by the intrinsic content and composition of polyphenols, determined by raw material attributes, such as origin, particle size, and extraction method [20,21,22]. While studies have examined processing effects and some macronutrient interactions, a systematic framework linking polyphenol preparation, molecular interactions, and product quality remains lacking. To fill this gap, the present study integrated internal and external factors in noodles and applied quantitative modeling to establish predictive relationships between *R. roxburghii* pomace particle size and key quality indicators.

Therefore, this study systematically investigated the effects of *R. roxburghii* pomace particle size (0.1–250 μm) across five distinct ranges (250, 60–80, 20–40, 1–10, and 0.1–1 μm) on noodle systems. We tracked polyphenol extracts derived from each particle size fraction through both fresh and boiled noodles, analyzing key performance indicators, including texture, color, cooking characteristics, polyphenol retention and composition, starch digestibility, and antioxidant activity. Mathematical modeling was employed to establish quantitative “particle size–property” relationships and develop predictive models. This provides a theoretical basis for optimizing noodle quality and functionality through precise particle size control. Subsequently, correlation analysis identified critical phenolic components responsible for the observed effects. Finally, we probed the molecular interactions of these key phenolic compounds with glutenin and starch within the processed noodle system, elucidating the fundamental mechanisms underlying the macroscopic property changes.

## 2. Materials and Methods

### 2.1. Material and Chemicals

*Rosa roxburghii* Tratt pomace was provided by the Guizhou Institute of Biotechnology (Guizhou, China). α-Amylase (*Bacilus subtilis*, 50 U/mg) was purchased from Shanghai Yuanye Biotechnology Co., Ltd. (Shanghai, China). Alkaline protease (≥200 U/mg), PBS phosphate-buffered saline solution (pH 7.1–7.3), DPPH (≥97.0%), potassium chloride (≥99.5%), and pepsin (≥1200.0 U/g) were purchased from Nanjing Dolay Biotechnology Co., Ltd. (Nanjing, China). Sodium hydroxide, 95% ethanol, acetone, ethyl acetate, n-hexane, and methanol (all analytical grade) were purchased from Chengdu Kelong Chemical Reagent Co., Ltd. (Chengdu, China). Concentrated hydrochloric acid (superior grade) was purchased from Chongqing Wansheng Chuandong Chemical Co., Ltd. (Chongqing, China). Standards for hydroxybenzoic acid, rutin, EGCG, catechin, epicatechin, isoquercitrin, ferulic acid, and quercetin were purchased from Sigma-Aldrich Co. (St. Louis, MO, USA).

### 2.2. The Preparation of Rosa roxburghii Pomace with Different Particle Sizes

The pomace, provided by the juice manufacturer as processing by-product, was subsequently dried to constant weight before use. The dry *Rosa roxburghii* Tratt pomace was initially pulverized using a high-speed multifunctional grinder (Model CX-A1500, Shanghai Yuanwo Industrial & Trade Co., Ltd., Shanghai, China) at 25,000 rpm for 3 min, producing powder with a particle size of 50–300 mesh, and then passed through a 60-mesh sieve to obtain the primary *Rosa roxburghii* Tratt pomace powder (250 μm).This primary residue powder was then used as the raw material and was subjected to micronization using a low-temperature high-energy nano-impact mill Model CXM-4 managed by Taiji Ring Nano Material Research Institute (Qinhuangdao City, China). Milling was performed at 380 rpm with a 4 L 304 stainless steel grinding chamber, using zirconia beads (6, 8, 10, 12, 15 mm) as the grinding medium, under continuous cooling at 8 °C for 6 h. This treatment yielded powders with particle sizes of 0.1–1 μm, 1–10 μm, 20–40 μm, and 60–80 μm, respectively. The particle size distribution of the fractions was determined using a laser diffraction particle size analyzer, ensuring accurate classification

### 2.3. The Preparation of Phenolic Extract

Following a previous report [26], 5.0 g of sample was mixed with cold 80% acetone (*v*/*v*), homogenized in an ice bath for 2 min, and centrifuged (3500 r/min, 10 min). The extraction was repeated twice on the residue and supernatants combined. After vacuum filtration, the solution was concentrated by rotary evaporation (45 °C) and freeze-dried to obtain the free phenol extract. The residue was then hydrolyzed with 20 mL 2 M NaOH in the dark for 1.5 h, neutralized, washed with 40 mL n-hexane (discarding the hexane layer), and extracted five times with ethyl acetate (40 mL each). The ethyl acetate extracts were combined, concentrated (rotary evaporation, 45 °C), washed with distilled water, and freeze-dried to yield the bound phenol extract. Extracts were characterized by total phenolic content (TPC, mg GAE/g DW). The final phenolic extract sample was prepared by combining the freeze-dried free and bound extracts. To ensure sample safety, all organic solvents (acetone, n-hexane, ethyl acetate) were completely removed by rotary evaporation and freeze-drying, and the final phenolic extracts used for noodle preparation contained no residual solvents.

### 2.4. The Preparation of Noodles

**Figure 1:**
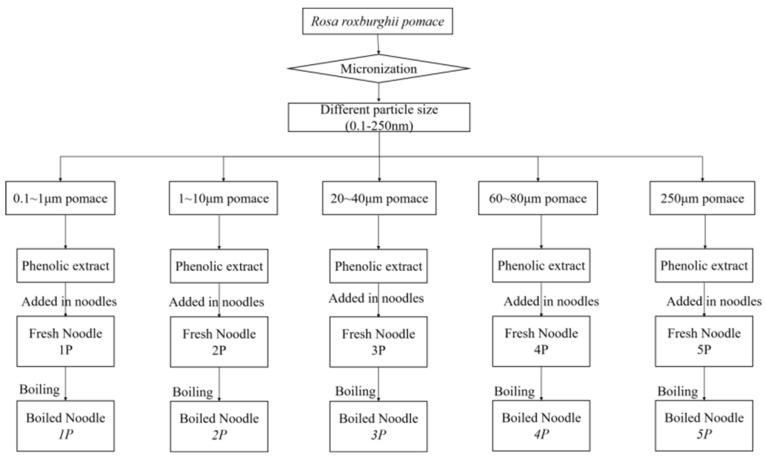
The procedure for preparing the noodle samples.

A total of 3 g of phenolic extract (free + bound fractions, prepared as described in Section 2.3) was incorporated into 97 g of wheat flour, corresponding to 3% (*w*/*w*, flour basis). This fixed ratio was consistently applied across all particle size treatments, while the control group contained no added extract. Salt and water were added (flour/salt/water = 10:1:4), and the dough was kneaded for 10 min, then rested for 15 min. Noodles were prepared using a small dough roller, with care taken to minimize dough/phenolic loss during kneading, pressing, and cutting. The noodles were divided: one portion for raw noodle analysis, the other for boiling tests. See Figure 1 for the procedure and Table 1 for sample labels.

### 2.5. Total Phenolic Content (TPC)

The TPC of the noodles was detected following the method reported by Adom and Liu [27] with slight modifications. Briefly, a gallic acid stock solution was precisely prepared at a concentration of 1 mg/mL. This stock solution was subsequently diluted to generate a series of standard solutions with concentrations ranging from 0 to 600 μg/mL. For each standard solution, 0.2 mL was taken and diluted with distilled water to a total volume of 1 mL. Then, 0.2 mL of Folin–Ciocalteu reagent was added, and the mixture was allowed to stand for 6 min. After this, 2 mL of 7% Na_2_CO_3_ solution and 1.6 mL of deionized water were added and mixed thoroughly. The solution was then incubated in the dark for 90 min. Absorbance was measured at a wavelength of 760 nm. The results were expressed as gallic acid equivalents per gram of dry noodle sample (mg GAE/g dry-weight basis, mg GAE/g DW). Noodles were oven-dried at 60 °C until constant weight, with a final moisture content of approximately 11–12%, and all polyphenol data were expressed on a dry-weight basis.

### 2.6. HPLC Analysis of Phenolic Components

The standards of phenolic compounds, including hydroxybenzoic acid, EGCG, catechin, and epicatechin, were dissolved in chromatographic-grade methanol to prepare standard solutions with concentrations ranging from 6.25 to 100 µg/mL. The vacuum freeze-dried free and bound phenol extract was firstly dissolved in chromatographic-grade methanol and then subjected to centrifugation (4000 r/min, 4 °C, 10 min). The resulting supernatants were diluted ten-fold with methanol, and then filtered through a 0.22 µm microfilter membrane and subsequently utilized for HPLC analysis, which was conducted using an Agilent 1260 Infinity II HPLC system (Agilent Technologies, Inc., Santa Clara, CA, USA) equipped with a DAD detector. The chromatographic separation was achieved on an Agilent ZORBAX Eclipse Plus C18 column (4.6 mm × 150 mm, 4 µm) (Agilent Technologies, Inc., USA). The flow rate was set at 0.4 mL/min, with the column temperature maintained at 35 °C. The injection volume was 20 µL. The mobile phases consisted of 0.1% glacial acetic acid in water (phase A) and acetonitrile (phase B). The gradient elution program was as follows: 0–10 min, 5% B; 30 min, 30% B; 40 min, 50% B; 50 min, 100% B; 55 min, 100% B; 60 min, 5% B; and 65 min, 5% B. Chromatographic data were recorded across a wavelength range of 190–800 nm.

### 2.7. Color

Color analysis of noodles was performed according to the CIELAB color space [28], using L*, a*, and b* coordinates. The color difference of the noodles was measured using an UltraScan PRO color measurement instrument (HunterLab, Reston, VA, USA). After preheating and calibrating the instrument, the prepared noodles were laid flat on a blank sheet of paper. The L*, a*, and b* values of the noodle samples were then detected. ΔE is calculated as shown in Equation (1):

(1) $$\Delta E\;=\sqrt{{\left(L_{n}-L_{0}\right)}^{2}+{\left(a_{n}-a_{0}\right)}^{2}+{\left(b_{n}-b_{0}\right)}^{2}}$$

where the subscript n refers to the sample after treatment, and 0 refers to the control sample.

### 2.8. Texture

Based on the method reported elsewhere [29], the texture property analysis (TPA) was conducted using the TA.XT-type texture analyzer (Stable Micro Systems, Godalming, UK). The noodles were boiled for 5 min, then immediately placed into cold water for 2 min to cool. After cooling, the noodles were drained on a sieve for about 15 s. Before testing, the probe return height was calibrated to 5 mm. The pre-test, test, and post-test speeds were all set to 0.8 mm/s, with a compression ratio of 70%. The interval between the two compression operations was set to 10 s, with a trigger force of 5 g. Each sample was tested six times in repetition.

### 2.9. Boiling Properties

The cooking loss (CL) of noodle samples was determined based on the method reported before [30] with slight modifications. The water absorption (WA) was determined by calculating the weight gain ratio based on raw noodle solids [31]. The CL and WA are calculated using Equations (2) and (3), respectively:

(2) $$\mathrm{CL}=\frac{m_{3}-m_{4}}{m_{3}}\times 100\%\;$$

(3) $$\mathrm{WA}=\frac{{\mathrm{CL}+m}_{2}-m_{1}}{m_{1}}\times 100\%$$

where *m*_1_ represents the mass of the raw noodles; *m*_2_ represents the mass of the boiled noodles; *m*_3_ represents the dry mass of the raw noodles; and *m*_4_ represents the dry mass of the boiled noodles.

### 2.10. Antioxidant Activity

DPPH radical scavenging activity was determined following the method reported by Juárez-Trujillo et al. [32] with slight modifications. The phenolic extract was mixed with DPPH solution and then placed in the dark for 30 min, after which the absorbance of the mixture was measured at 517 nm. The DPPH radical scavenging activity was calculated using Formula (4):

(4) $$\mathrm{DPPH}\;\mathrm{radical}\;\mathrm{scavenging}\;\mathrm{activity}\left(\%\right)=\frac{A_{o}-\left(A_{s}-A_{c}\right)}{A_{o}}\times 100\%$$

where *A_o_* represents the absorbance of a mixture of 2 mL of DPPH solution and 2 mL of ethanol; *A_c_* represents the absorbance of 2 mL of sample solution mixed with 2 mL of ethanol; *A_s_* represents the absorbance of 2 mL of 0.1 mmol/L DPPH ethanol solution mixed with 2 mL of noodle extract; and *m*_3_ represents the dry mass of the raw noodles.

### 2.11. Starch Digestion

#### 2.11.1. Total Starch Content

The determination of total starch content was conducted according to the enzymatic hydrolysis method described in Chinese National Standards GB 5009.9-2016 [33], with slight modifications. Briefly, approximately 0.10 g of the dried and ground noodle sample (dry basis) was dispersed in distilled water and subjected to liquefaction with thermostable α-amylase (95–100 °C, 30 min) to hydrolyze gelatinized starch. After cooling, the pH was adjusted to 4.5–4.8, followed by saccharification with amyloglucosidase (60 °C, 30–60 min) to convert starch into glucose. The reaction was terminated by boiling, and the hydrolysate was centrifuged to obtain the supernatant. The reducing sugar content of the supernatant was determined using the 3,5-dinitrosalicylic acid (DNS) method, with reference to a glucose standard curve. The calculation formula for total starch content was given by Equation (5):

(5) $$\mathrm{Total}\;\mathrm{Starch}\;\mathrm{Content}\;\left(\%\right)\;=\;\mathrm{Glucose}\;\mathrm{equivalent}\;\left(\%\right)\;\times \;0.9$$

#### 2.11.2. The Simulated Digestion In Vitro

Following the method reported by Sharavathy et al. [34] with slight modifications, briefly, the boiled noodles were manually ground for one minute to simulate mastication. The homogenized noodle sample was then mixed with 5 mL of simulated saliva, prepared using an α-amylase (50 U/mg, Shanghai Yuanye Biotechnology Co., Ltd., Shanghai, China) solution at a concentration of 200 U/mL in PBS (pH 7.2). The mixture was incubated in a shaking water bath at 37 °C for 5 min. After incubation, the pH of the mixture was adjusted to 1.5 using HCl-KCl buffer (pH 1.5), and 1 mL of pepsin (≥1200 U/g, Nanjing Dolay Biotechnology Co., Ltd., Nanjing, China) solution (1 mg/mL) was added. The mixture was incubated in a shaking water bath at 40 °C for one hour. After gastric digestion, the sample was cooled to room temperature and the total volume was adjusted to 25 mL with PBS buffer (pH 6.9). Then, 2 mL of α-amylase solution (200 U/mL) was added and incubation was continued in a shaking water bath at 37 °C to simulate small intestinal digestion. At digestion times of 0, 30, 60, 90, 120, 150, and 180 min, 1 mL of the digestion solution was withdrawn, and the enzyme activity was terminated by placing the samples in a boiling water bath for 6 min. The reducing sugar content was determined using the DNS method, and the starch digestion rate was calculated according to Formula (6):

(6) $$\mathrm{Starch}\;\mathrm{Digestibility}\;(\%)\;=\;\frac{\left(\mathrm{Glucose}\;\mathrm{Equivalent}\;\times 0.9\times 100\right)}{\mathrm{Total}\;\mathrm{Starch}\;\mathrm{Content}}$$

### 2.12. Molecular Docking

The molecular docking was performed according to a published procedure with slight modifications [35]. The structure of glutenin was obtained from the AlphaFold Protein Structure Database (PDB ID: AF_AFP08488F1) (https://alphafold.ebi.ac.uk) [accessed 25 June 2025]. Receptor proteins were pre-processed by removing water molecules, adding polar hydrogens, and assigning Gasteiger charges using AutoDock Tools Version 1.5.7 [36]. The three-dimensional structures of five phenolic compounds—gallic acid (CID: 370), EGCG (CID: 65064), hydroxybenzoic acid (CID: 135), quercetin (CID: 5280343), and isoquercitrin (CID: 5280804)—were retrieved from the PubChem database (https://pubchem.ncbi.nlm.nih.gov) [accessed on 25 June 2025] and converted into PDBQT format using Open Babel. Molecular docking was conducted using AutoDock Vina Version 1.2.5 [37], with a grid box covering the entire ligand–receptor complex. Each ligand was docked to both receptor structures, and binding affinity values were recorded. Docking results were analyzed and visualized using PyMOL Version 3.0.2, LigPlot+ Version 2.2.8 and PLIP (https://plip-tool.biotec.tu-dresden.de/plip-web/plip/index) [accessed on 25 June 2025].

### 2.13. Statistical Analysis

All experiments were conducted in triplicate, and all quantitative data are presented as mean values ± standard deviations (SD). Prior to statistical testing, data normality and homogeneity of variances were examined to ensure the validity of parametric analyses. One-way analysis of variance (ANOVA) was performed using SPSS 23.0 software (SPSS Inc., Chicago, IL, USA), with statistical significance accepted at *p* < 0.05. When significant effects were observed, Tukey’s HSD post hoc test was applied for pairwise multiple comparisons (*p* < 0.05). Statistically distinct groups are denoted by different superscript letters in the tables and figures. All graphs and regression plots were generated using Origin 2021 software (OriginLab Corporation, Northampton, MA, USA).

## 3. Results and Discussion

### 3.1. Total Phenolic Content (TPC) of Noodles

Although the amount of crude phenolic extract added to the noodles remained constant at 3%, variations in the particle size of *Rosa roxburghii* Tratt pomace led to discrepancies in the release rates of the phenolic components during extraction, resulting in different TPCs of the noodles. Table 2 summarizes the TPCs of noodles enriched with the phenolic extracts both prior to and following the boiling process.

#### 3.1.1. TPC of the Fresh Noodles

The TPC of fresh noodles decreased in the order 2P > 1P ≈ 3P ≈ 4P > 5P (Table 2), indicating that smaller pomace particle sizes (1–10 μm: 2P) generally enhanced phenolic release compared to larger sizes (20–250 μm: 3P, 4P, 5P). *Rosa roxburghii* Tratt pomace itself is rich in phenolic acids (e.g., gallic acid, p-hydroxybenzoic acid, chlorogenic acid, ferulic acid) and flavonoids (e.g., rutin, quercetin, isoquercitrin, catechin, and epicatechin) [2], many of which were also detected in our noodle samples. 

Reduced particle size increased surface area, improving solvent access and releasing trapped phenolics. Similarly, the polyphenol content of olive pomace (15.6 μm) was consistently higher than that of the pomace with a particle size of 17.8 μm [38]. Micronization treatment helped to convert bound polyphenols into free forms, which allowed for more efficient extraction and utilization of the polyphenols [21]. However, excessively small particles (<1 μm, e.g., 1P) showed lower TPC than moderately small particles (1–10 μm, e.g., 2P). This is attributed to particle agglomeration impeding solvent penetration during the extraction of polyphenols from pomace [20] and potential heat degradation during intensive micronization of pomace [22]. An optimal particle size range existed for phenolic extraction, as supported by findings that moderately reduced, uniform sizes (e.g., 16–18 μm) maximized release efficiency [39].

Thus, smaller pomace particles (0.1–10 μm) outperformed larger sizes (20–250 μm) in releasing phenolics, and the most favorable size range for *Rosa roxburghii* pomace was 1–10 μm rather than 0.1–1 μm.

#### 3.1.2. TPC of the Boiled Noodles

The TPC of all boiled noodles was significantly lower than that of fresh noodles (Table 2), and polyphenol retention ranged from 8.5% to 16.7%. Samples *3P*, *4P*, and *5P* showed minimal variation, averaging 11.5% retention. *1P* exhibited the highest retention (16.72%). *2P*, despite its highest initial TPC, showed the lowest retention (8.51%). This substantial polyphenol loss might be primarily due to leaching into boiling water [40], thermal degradation [41], and oxidation [42]. Boiling likely caused polyphenols to cross-link with proteins or starch, encapsulating them and lowering the detectable TPC [43,44].

The variation in TPC among samples may stem from their distinct polyphenol compositions, as different polyphenols exhibited differential heat sensitivity, and some degraded preferentially under the same conditions [41]. Notably, although 2P showed the highest initial TPC, it exhibited the lowest retention after boiling, which could be linked to its compositional features: compared with 1P, 2P was initially enriched in HBA, IQ, and FA, but these compounds declined sharply during cooking (e.g., FA decreased from 486.82 μg/g to 15.30 μg/g). Such differences in polyphenol composition likely contributed to the paradox of low retention despite high initial TPC, although the precise mechanisms remain to be further verified. Therefore, HPLC was employed to specifically analyze the polyphenol profile of each noodle sample.

### 3.2. Individual Phenolic Content of Noodles

**Figure 2:**
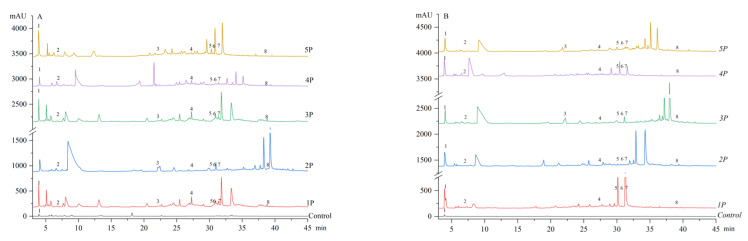
The HPLC chromatograms of the fresh noodles (**A**) and boiled noodles (**B**). The identified phenolic compounds include (1) gallic acid (*Rt.* = 4.04 min), (2) hydroxybenzoic acid (*Rt.* = 6.96 min), (3) EGCG (*Rt.* = 22.12 min), (4) epicatechin (*Rt.* = 22.41 min), (5) isoquercitrin (*Rt.* = 30.26 min), (6) ferulic acid (*Rt.* = 30.75 min), (7) rutin (*Rt.* = 31.18 min), and (8) quercetin (*Rt.* = 38.88 min).

HPLC analysis identified eight phenolic compounds (gallic acid, hydroxybenzoic acid, EGCG, epicatechin, isoquercitrin, ferulic acid, rutin, quercetin) in both fresh and boiled noodles (Figure 2, Table 3).

In fresh noodles, gallic acid showed the lowest content, likely due to its low extraction efficiency from pomace and strong binding (e.g., forming stable V-type complexes with starch via hydrophobic interactions) or encapsulation within the noodle matrix (proteins/starch), impeding its release and detection [45,46]. Polyphenol profiles varied significantly among fresh noodle samples (TPC: 366.24 μg/g in 3P to 1199.64 μg/g in 5P), probably attributed to pomace particle size effects on extraction, as illustrated in Section 3.1.1.

Boiling induced qualitative and quantitative changes; that is, EGCG disappeared in *1P*/*2P* while it likely appeared in *4P*, consistent with *2P*’s substantial TPC loss (Section 3.1.2). The degradation of phenolic compounds probably resulted from the breaking of covalent bonds or enhanced oxidative reactions due to thermal treatment or oxidase-catalyzed reactions [47,48]. Among phenolic compounds, flavonoids were particularly susceptible to boiling [41]. Typically, heat-sensitive compounds such as EGCG and epicatechin (EC) are more prone to degradation and structural modifications under high-temperature conditions. For example, it was reported in previous studies that EGCG could be thermally degraded into GCG or EGCG dimers [49]. It may undergo epimerization or dimerization under high-temperature conditions, but such mechanisms were not directly verified in this study. EC can isomerize into catechin, with the trans form being thermodynamically more stable than the cis form [50]. The oxidation tendency followed the order of EGCG > EGC > EC [51,52]. This may help explain the pronounced reduction in EGCG observed in 1P and 2P. However, transformation also occurred, with hydroxybenzoic acid significantly decreasing in *1P*/*2P*, which could be associated with changes in gallic acid content. Meanwhile, EGCG and ferulic acid increased in *3P*/*4P*, and hydroxybenzoic acid increased in *5P* alongside a decrease in gallic acid, suggesting that gallic acid may have been converted to hydroxybenzoic acid as an intermediate or product of degradation [53].

These dynamic fluctuations probably stemmed from inherent variations in phenolic thermal stability triggering interactions/degradation and complex reactions between phenolics and noodle matrix components (starch/protein) during boiling and gelatinization, leading to both degradation of the original compounds and formation of new species or derivatives. Similarly, during ultra-high-temperature processing, EGCG underwent epimerization into gallocatechin gallate (GCG), which further reacted with α-dicarbonyl intermediates or hydroxymethylfurfural to form novel phenolic derivatives [54]. During gelatinization of sorghum, phenolic compounds significantly interacted with both starch and protein fractions, ultimately affecting the composition and concentration of detectable polyphenols [55,56]. While these interactions and thermal processes clearly alter the phenolic profile, the precise mechanisms governing the net “formation” versus “degradation” require further elucidation.

However, this study did not include a systematic characterization of the chemical composition of pomace across different particle sizes. Although HPLC confirmed the presence of key polyphenols and previous studies have reported the overall composition of *Rosa roxburghii* pomace, potential particle size-dependent variations in minor compounds remain unclear.

### 3.3. Color of Noodles

**Figure 3:**
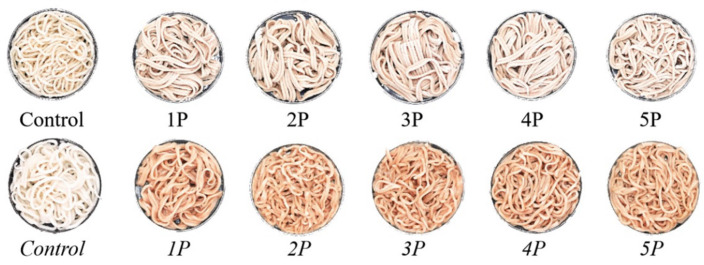
Images of the fresh and boiled noodles with the addition of polyphenol extracts. The sample numbers are consistent with those shown in Table 1.

Color analysis (Table 4, Figure 3) revealed that incorporating pomace polyphenols significantly altered noodle color.

Both the fresh and boiled noodles exhibited decreased lightness (L*), increased red/green intensity (a*), and increased yellow/blue intensity (b*) compared to the controls, indicating darker noodles with intensified red and yellow hues, primarily attributed to pomace pigments and polyphenols [57]. Boiling further reduced L* values in all samples relative to their fresh counterparts, likely due to thermal degradation of compounds, polyphenol leaching into the cooking water, and persistent enzymatic browning involving polyphenol oxidase (PPO) activity and interactions between degraded polyphenols and generated pigments [58,59,60]. The color variations in the noodles were positively correlated with the levels of flavonoids, polyphenols, and other pigment substances [61]. Additional critical determinants included temporal factors, thermal regimes, processing variables (e.g., water absorption), flour protein content, milling techniques, and processing–environment interactions [62].

Accordingly, the incorporation of the pomace polyphenols significantly darkened both the fresh and boiled noodles (reduced L*) while intensifying red and yellow hues (increased a*, b*).

### 3.4. Texture Profile

**Figure 4:**
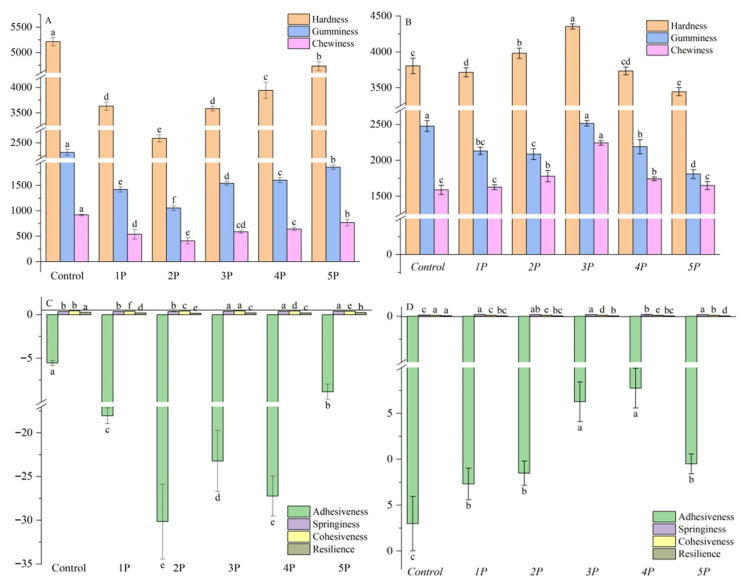
The TPA parameters of the fresh (**A**,**C**) and boiled (**B**,**D**) noodles containing the polyphenol extracts. Texture profile analysis parameters of noodles with different pomace particle sizes. Hardness and gumminess are expressed in Newtons (N), chewiness in N·mm, and adhesiveness in N·s, while springiness, cohesiveness, and resilience are dimensionless. Different letters in the same row donate significant differences (*p* < 0.05).

Texture profile analysis (Figure 4) showed that incorporating phenolic extracts reduced hardness, gumminess, chewiness, and adhesiveness in fresh noodles compared to the controls, with samples 2P and 3P exhibiting the most significant reductions; springiness, cohesiveness, and resilience remained unchanged.

A noodle is characterized by starch granules embedded in a well-developed gluten network [63]. The weakening of the gluten network likely resulted from polyphenols disrupting disulfide bond cross-linking via antioxidant activity [64] and forming starch–polyphenol complexes (e.g., V-type inclusions through hydrophobic interactions), which reduced starch crystallinity and altered water distribution, thereby decreasing hardness [23,24,25]. This disruptive effect was particularly evident in fresh noodles prior to thermal processing. It therefore could be inferred that the 2P and 3P noodles exhibited the most significant reductions in TPA parameters **(**Figure 4A,C), which may be due to their higher phenolic contents.

After boiling, a phase shift in interactions occurred; conversely, boiled phenolic-enriched noodles (notably *2P*/*3P*) exhibited increased chewiness and adhesiveness. Boiling likely strengthened the gluten network through new protein cross-links and induced covalent bonding between thermally oxidized polyphenols (forming quinones) and unfolded proteins, promoting aggregation and network stability [18]. Additionally, polyphenols may enhance protein solubility and matrix interactions, further contributing to increased chewiness [65]. *2P* and *3P* showed increased TPA parameters (Figure 4B,D), which might be because noodles with added phenolic extract probably formed a well-organized gluten network, enhancing dough stability and extensibility, indicating that polyphenols may act as destabilizers in fresh noodles but stabilizers after heat treatment.

The pronounced texture changes in these noodles (especially 2P/3P and *2P*/*3P*) correlate with their higher phenolic content, demonstrating that pomace particle size influences texture primarily through the levels of polyphenol extracts and their interactions with starch/protein components.

### 3.5. Cooking Properties

**Figure 5:**
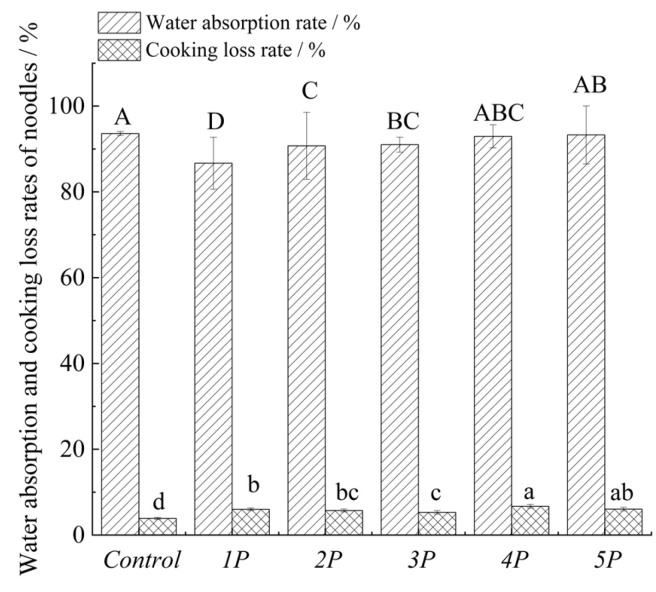
Influence of the addition of the phenolic extract on the water absorption and loss rates of boiled noodles. The sample number is consistent with Table 1. For the same index, different letters among sample groups indicate significant differences (*p* < 0.05).

The cooking properties of noodles were expressed as water absorption (WA) rate and cooking loss (CL) rate, and the results are shown in Figure 5.

#### 3.5.1. Water Absorption Rate

Water absorption during boiling involves infiltration from the surface to the core, hindered by starch gelatinization and protein denaturation densifying the gel matrix and restricting internal space [66]. Compared to the controls, WA significantly decreased in noodles with phenolic extracts (*1P*–*3P*), with *1P* (highest TPC, Table 2) exhibiting the lowest WA (Figure 5). This reduction probably stemmed from polyphenol-induced disruption of gluten networks via disulfide bond cleavage, loosening the structure and exposing starch granules. Additionally, starch–polyphenol hydrogen bonding competed with water–starch interactions, while extensive polyphenol–protein cross-linking overrode hydration effects, collectively impeding water uptake [46,67,68]. Conversely, *4P* and *5P* showed WA rates equivalent to the controls, likely due to compensatory mechanisms. That is, polyphenols’ hydrophilic hydroxyl groups enhanced water retention, partially offsetting their competition with proteins for water and starch–polyphenol binding that altered hydration dynamics [24,43,69]. *4P* and *5P* exhibited WA rates similar to the control, likely due to combined polyphenol interactions with water, starch, and protein—alongside starch–protein dynamics. This complexity warrants further study.

#### 3.5.2. Cooking Loss (CL)

Cooking loss refers to the amount of moisture, soluble substances, and nutrients lost during boiling. All phenolic-enriched noodles exhibited higher CL than the controls (Figure 5), indicating increased leaching of solubles during boiling. CL peaked in *4P*, correlating with its highest polyphenol content (Table 3) and hydrophobic compound prevalence (e.g., ferulic acid, Table 3), which excessively formed starch–polyphenol complexes that disrupted the continuous gel network and weakened intermolecular starch interactions [46,69,70]. Polyphenol hydrophobicity/polarity appeared to influence CL: hydrophobic types (e.g., ferulic acid, epicatechin) tended to promote starch leaching, while polar polyphenols (e.g., rutin, hydroxybenzoic acid) were more likely to stabilize the gel network through hydrogen bonding [71]. Consequently, 2P (with relatively high hydroxybenzoic acid but dominated by ferulic acid) showed elevated CL, whereas 3P (enriched in more polar phenolics such as hydroxybenzoic acid and rutin) exhibited lower CL. Despite 1P’s high rutin content, its abundant hydrophobic disruptors (epicatechin, ferulic acid) drove the highest CL.

It should be emphasized that this interpretation is qualitative, focusing on the presence or absence of polarity rather than quantitative strength. The results suggest that phenolic polarity may alter the probability of interactions with starch–protein networks, indirectly influencing CL. Similar observations have been reported in studies linking polyphenol polarity with starch/protein [72,73]. However, no correlation tests (e.g., Pearson’s correlation) were conducted here to quantitatively validate the relationship, which has been acknowledged as a limitation of this study.

### 3.6. Antioxidant Ability

**Figure 6:**
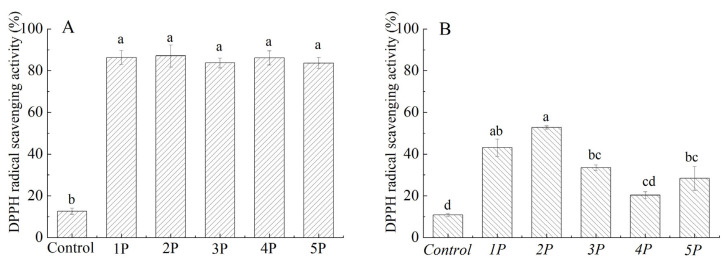
Influence of the addition of the phenolic extracts on the DPPH free radical scavenging capacity of the fresh noodles (**A**) and the boiled noodles (**B**). Different letters among sample groups indicate significant group differences (*p* < 0.05).

The antioxidant capacity of noodles was assessed via DPPH radical scavenging activity (Figure 6).

Fresh noodles with added phenolic extracts exhibited uniformly high DPPH activity (80% higher than controls, *p* < 0.05), despite variations in polyphenol content (Table 3). This consistent activity likely stems from the intrinsic radical scavenging ability of polyphenols (e.g., quercetin, epicatechin, gallic acid), which donated hydrogen atoms via phenolic hydroxyl groups to terminate oxidation chains [4]. Polyphenol interactions with starch or proteins may either retain radical scavenging activity or encapsulate antioxidant phenolic compounds, though the mechanisms require further study.

Boiling markedly reduced DPPH activity (average: ~35% vs. fresh noodles’ 85%). This decline primarily resulted from thermal degradation of polyphenols, leaching into cooking water, and loss of soluble antioxidants (e.g., free sugars, proteins) [74].

Notably, while 2P did not have the highest TPC level (Table 2, Table 3), it showed the highest DPPH radical scavenging activity among all the boiled noodles (Figure 6B). A plausible explanation for this is that certain structural modifications of polyphenols during heating, such as the epimerization of EGCG→EGC reported in previous studies [21,47,54], may yield derivatives with retained or even enhanced antioxidant activity. Another explanation is conformation-stabilizing interactions with the matrix. However, such mechanisms still need to be validated. Overall, phenolic extracts enhanced fresh noodle antioxidant capacity, but boiling-induced losses underscore the sensitivity of phenolic compounds to thermal processing.

### 3.7. Starch Digestion Rate

**Figure 7:**
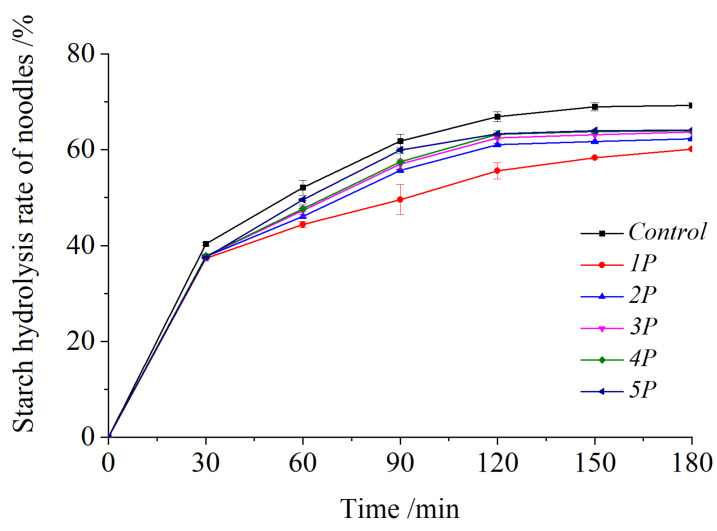
Effect of the addition of the phenolic extract on the starch hydrolysis rate of boiled noodles.

All phenolic-fortified noodles exhibited lower starch hydrolysis rates than the controls (Figure 7).

During the initial 30 min of digestion, hydrolysis rates were similar across samples, indicating limited early-stage inhibition by polyphenols. Subsequently, hydrolysis diverged, following the order *1P* < *2P* < *3P* < *4P* < *5P < control*. At 90 min, the inhibitory effect of sample *1P* was almost 1.45-fold higher than that of the *control* sample. This sequence did not strictly align with polyphenol content (Table 2 and Table 3), suggesting that additional factors influenced digestibility.

Reduced hydrolysis is attributed to polyphenols competitively inhibiting α-amylase/α-glucosidase via active-site binding [75,76,77], with efficacy dependent on polyphenol type, starch structure, and binding interactions [24]. In particular, polyphenols with multiple hydroxyl groups and conjugated aromatic rings (e.g., EGCG, quercetin) generally exhibit stronger inhibitory activity through hydrogen bonding and π–π stacking, whereas simpler phenolic acids (e.g., hydroxybenzoic acid, ferulic acid) exert relatively weaker inhibition [78]. Notably, the combination of different phenolic acids was more effective in inhibiting α-amylase compared to the individual phenolic acids [79]. The interactions between starch chains and phenolic compounds were also related to the decreased starch hydrolysis rates [80,81]. Furthermore, enzyme-resistant starch–polyphenol complexes formed through hydrogen bonding or electrostatic interactions physically hindered substrate accessibility and enzymatic breakdown [16,25], while dense network structures limited polyphenol release [82]. Collectively, phenolic extracts significantly suppressed starch hydrolysis, though mechanistic interplay with noodle components requires further investigation, which has potential commercial significance as reduced starch hydrolysis is associated with lower postprandial glycemic response. Such properties are highly desirable in the development of functional low-glycemic-index staple foods (e.g., noodles) for populations with diabetes, obesity, or metabolic syndrome, thereby enhancing both nutritional value and market potential.

However, all digestion assays were performed under in vitro conditions, which cannot fully replicate the complexity of the human gastrointestinal environment. Therefore, caution should be exercised when extrapolating these findings to in vivo contexts.

### 3.8. Correlation Analysis

**Figure 8:**
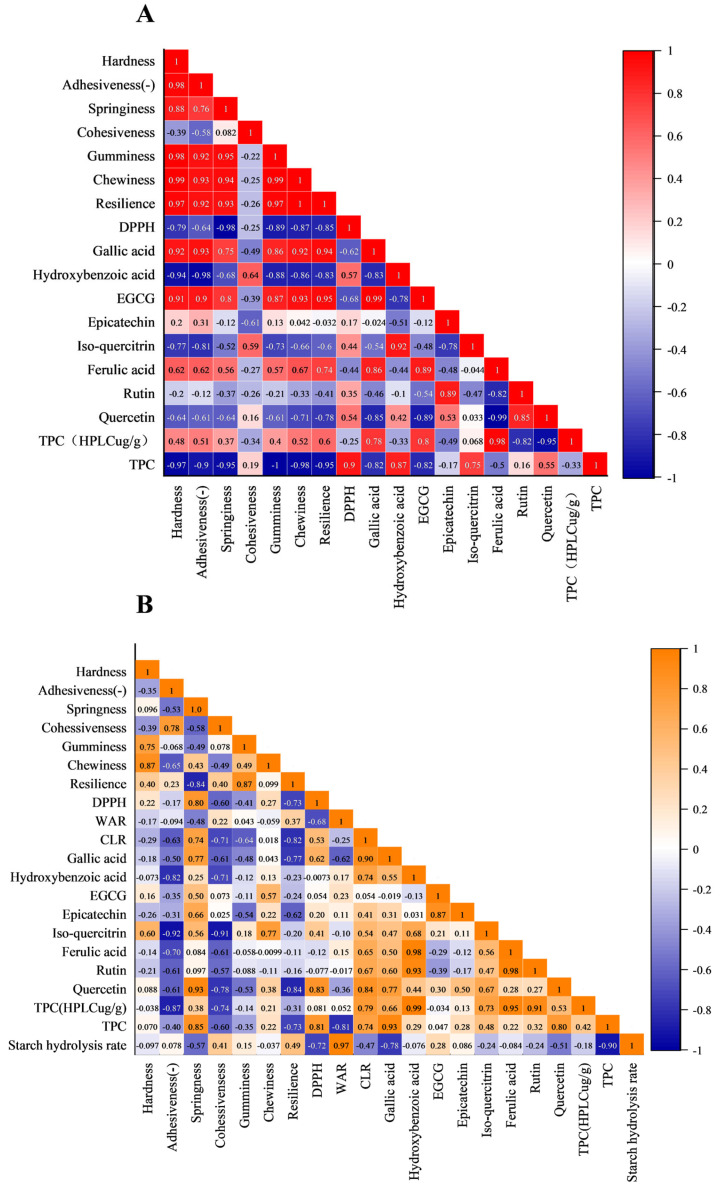
The correlation analysis of all the detected indexes of fresh noodles (**A**) and boiled noodles (**B**).

In fresh noodles (Figure 8A), hardness strongly correlated with adhesiveness (*r* = 0.98) and springiness (*r* = 0.76). Variables are expressed in their original units: hardness, gumminess, and chewiness: N; adhesiveness: N·s; springiness, cohesiveness, and resilience: dimensionless. The following substances were used: DPPH (µmol TE/g DW), gallic acid (GA), hydroxybenzoic acid (HBA), epicatechin (EC), EGCG, isoquercitrin (IQ), ferulic acid (FA), rutin, and quercetin (all µg/g DW), TPC (mg GAE/g DW), and TPC (HPLC, µg/g DW).

The texture parameters were negatively correlated with Folin TPC but positively correlated with HPLC TPC. Gallic acid and EGCG showed strong positive correlations (*r* = 0.75–0.95) with texture, suggesting that starch/protein interactions enhanced matrix integrity, whereas hydroxybenzoic acid exhibited negative correlations (*r* = −0.94 to −0.83), indicating disruptive effects. DPPH activity was negatively correlated with HPLC TPC (*r* = −0.25) but highly positively correlated with Folin-TPC (*r* = 0.90), implying synergistic antioxidant contributions from the combined action of all polyphenols, not just the eight identified ones shown in Figure 2. The synergistic effects among various polyphenols contributed more effectively to antioxidant capacity than the individual polyphenols [83]. The synergistic effects among polyphenols and other antioxidants (e.g., triterpenoids, polysaccharides) in *Rosa roxburghii* pomace may also contribute to scavenging DPPH [1,84,85,86].

After boiling, texture–polyphenol correlations weakened. Isoquercitrin was positively correlated with chewiness (*r* = 0.77) and hardness (*r* = 0.60) but negatively correlated with adhesiveness (*r* = −0.92) and cohesiveness (*r* = −0.91), while quercetin was positively linked to springiness (*r* = 0.93) and negatively linked to resilience (*r* = −0.84), indicating thermally altered polyphenol–starch/protein interactions. Gallic acid strongly inhibited starch hydrolysis (*r* = −0.78), contrasting with EGCG’s neutral effect (*r* = 0.28). Thermal degradation eliminated fresh noodle correlations (e.g., gallic acid/EGCG hardness), while isoquercitrin’s correlation with hardness was reversed (fresh: *r* = −0.77; boiled: *r* = 0.60) due to increased post-boiling levels in *1P*, *3P*, and *4P* (Table 3).

Accordingly, key polyphenols (gallic acid, EGCG, hydroxybenzoic acid, isoquercitrin, quercetin) influenced texture via starch/protein interactions, prompting further molecular docking analysis (Section 3.10).

### 3.9. Modeling Quality Parameter Dynamics in Noodles via Mathematical Fitting

Noodles incorporating phenolic extracts from *Rosa roxburghii* pomace (0.1–250 μm particle size) exhibited distinct quality properties. These variations originated from particle size-dependent differences in extract composition and concentration. Consequently, pomace particle size served as a predictor for noodle property modulation.

**Figure 9:**
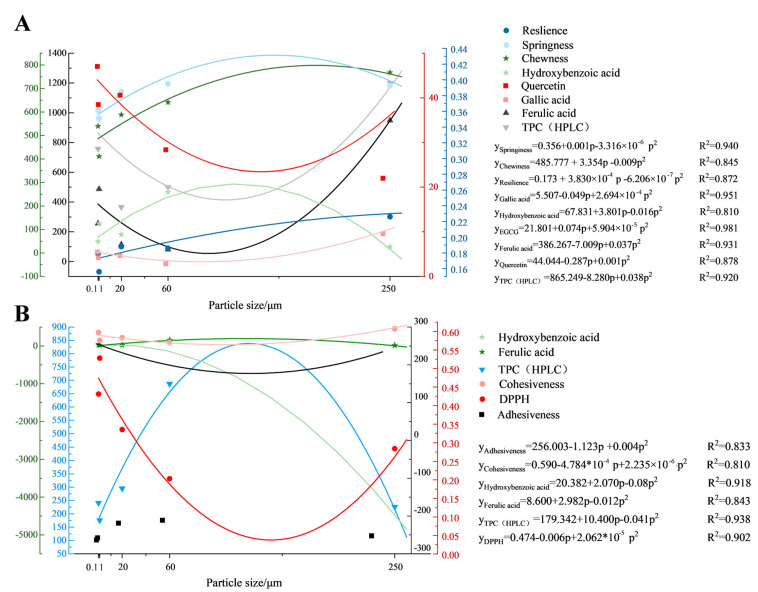
Quadratic regression curves for highly fitted detection parameters (R^2^ > 0.821) of fresh noodles (**A**) and boiled noodles (**B**). Variables are expressed in their original units. Hardness, gumminess, and chewiness: N. Adhesiveness: N·s. Springiness, cohesiveness, and resilience: dimensionless. DPPH (µmol TE/g DW). Gallic acid (GA), hydroxybenzoic acid (HBA), epicatechin (EC), EGCG, isoquercitrin (IQ), ferulic acid (FA), rutin, quercetin (all µg/g DW), TPC (mg GAE/g DW), and TPC (HPLC, µg/g DW).

Quadratic regression modeling (Figure 9, Supplementary Tables S1 and S2) revealed particle size-dependent modulation of noodle properties (0.1–250 μm pomace), where pomace particle size served as a predictor for extract-driven quality variations. Regression coefficients are presented with 95% confidence intervals. Goodness of fit was evaluated using R^2^ values.

In fresh noodles, particle size strongly predicted springiness (*R*^2^ = 0.94), chewiness (*R*^2^ = 0.845), and resilience (*R*^2^ = 0.872), but these relationships weakened after boiling (*R*^2^ = 0.151–0.642), except for boiled cohesiveness, which followed a distinct quadratic trend (y = 0.590 − 4.784 × 10^−4^p + 2.235 × 10^−6^p^2^, *R*^2^ = 0.821). Particle size reliably predicted phenolic profiles; that is, ferulic acid, hydroxybenzoic acid, and HPLC TPC exhibited high predictability in both fresh and boiled noodles (*R*^2^ = 0.810–0.938). The DPPH scavenging capacity in boiled noodles was accurately modeled (y = 0.474 − 0.006p + 2.062 × 10^−5^p^2^, *R*^2^ = 0.902), unlike in fresh noodles (*R*^2^ = 0.426), highlighting thermal processing as a critical modulator of particle size effects.

### 3.10. Molecular Docking Analysis of Interactions Between Phenolic Compounds and Glutenin/Starch

According to the correlation analysis (Figure 8), EGCG, hydroxybenzoic acid, gallic acid, quercetin, and isoquercitrin are closely correlated with noodle texture, probably resulting from joint interactions with glutenin and starch. Therefore, molecular docking simulations evaluated the interactions with both glutenin (a major structural protein) and starch (a key component governing texture). To better contextualize these simulations, the determinations of texture parameters (hardness, gumminess, chewiness, adhesiveness) and starch digestibility assays provided experimental evidence of polyphenol-mediated modifications in noodle structure and hydrolysis behavior. 

**Figure 10:**
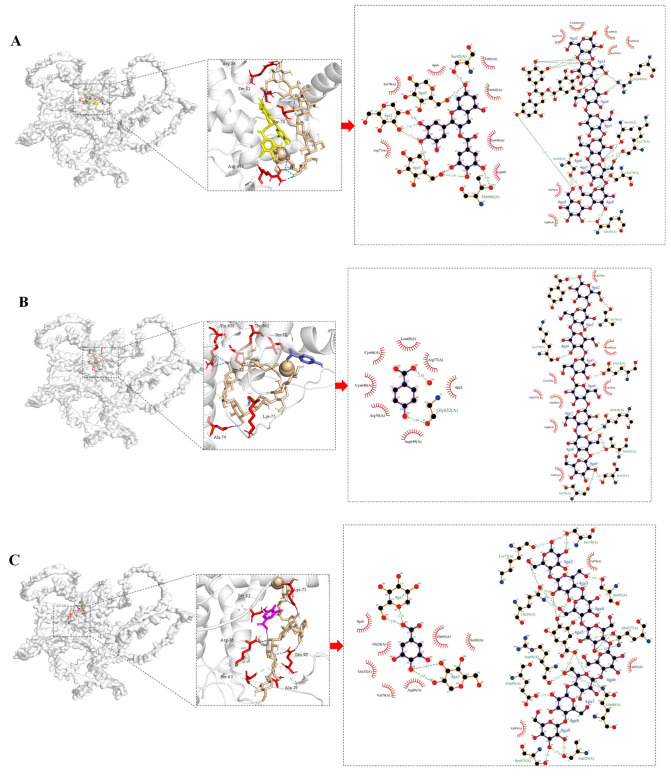

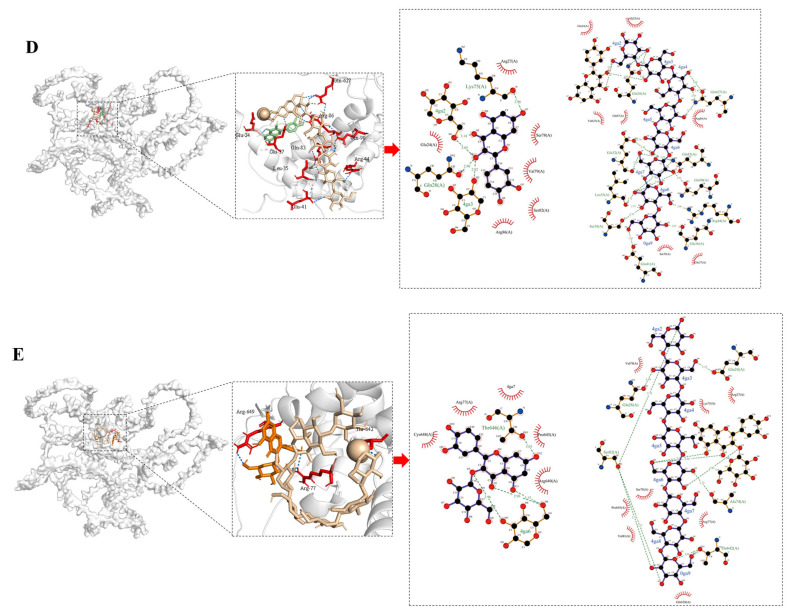
The molecular docking analysis of EGCG (**A**), hydroxybenzoic acid (**B**), gallic acid (**C**), quercetin (**D**), and isoquercitrin (**E**) with both glutenin and starch.

The molecular docking visualizations in Figure 10 and the binding sites summarized in Table 5 collectively demonstrate that all tested phenolic compounds spontaneously form stable ternary complexes with glutenin and starch, as evidenced by their negative binding energies ranging from −7.2 to −8.4 kcal/mol.

These complexes exhibit diverse molecular interactions, including hydrogen bonds, hydrophobic contacts, π–cation interactions, and salt bridges, with distinct spatial distribution patterns.

Glutenin–starch binding is predicted to be predominantly mediated by hydrogen bonding, which may govern the conformational stability of glutenin–starch assemblies—a critical determinant of dough texture [87]. The binding sites between glutenin and starch are extensively distributed across polar regions of the glutenin surface, including residues such as Ser-82, Ser-78, Gln-638, and Glu-24, which are key anchoring sites for stable starch–glutenin network formation and play essential roles in maintaining the integrity of the starch–glutenin complex.

Glutenin–phenolic interactions are suggested to be primarily driven by hydrophobic interactions, π–cation interactions, and salt bridges, with more diverse interaction types than in glutenin–starch binding. Quercetin exhibited the strongest binding affinity (−8.4 kcal/mol) to glutenin among phenolics, consistent with its documented capacity to form stable non-covalent complexes with gliadin (a structurally similar glutamine-rich protein) through multimodal interactions [88]. The binding energy between EGCG and glutenin reached -8.1 kcal/mol, demonstrating significant affinity primarily driven by hydrophobic interactions (Table 5). This binding relied on a multipoint hydrogen bond network formed between EGCG’s phenolic hydroxyl groups and the surface amino acid residues of the protein, complemented by hydrophobic interactions involving EGCG’s aromatic rings and hydrophobic residues (Figure 10A). These complex interactions promoted local compaction and enhanced the overall stability of the glutenin structure [89]. The *3P* group with higher EGCG content exhibited significantly greater hardness and chewiness (Table 3, Figure 4B), suggesting that EGCG–glutenin interactions may contribute to improved textural strength and deformation resistance in noodles. An affinity also existed between hydroxybenzoic acid and glutenin (binding energy ≈ −7.2 kcal/mol), yet the limited hydrogen-bonding sites and hydrophobic interaction points (Figure 10B) precluded dense protein–polyphenol network formation—unlike EGCG-type polyphenols. Phenolic acids could reduce flour product gumminess/chewiness through competitive water binding and starch–phenolic complexation, thereby disrupting starch chain interactions, altering starch architecture, and ultimately compromising protein–starch network integrity [56,90]. Consequently, the diminished gumminess in *4P* and *3P* (Table 3, Figure 4B) may primarily be attributed to their elevated hydroxybenzoic acid content.

The common interaction sites for the glutenin–polyphenol–starch ternary complex occurred at Arg-86 and Arg-649, which recurred across the complexes involving EGCG, quercetin, isoquercitrin, and gallic acid. Arg-86 functioned as a structural hub, forming hydrogen bonds with starch while engaging in π-stacking or electrostatic interactions with phenolics like gallic acid (Figure 10C)—sometimes inducing steric occlusion of binding sites. In contrast, Arg-649 preferentially formed salt bridges, exemplified by its guanidinium–carboxyl interaction with isoquercitrin. Such salt bridges represented exceptionally stable electrostatic interfaces essential for maintaining macromolecular conformational integrity [91]. Isoquercitrin formed stable salt bridges coupled with π–cation synergism at surface sites including Arg-649 (Figure 10E, Table 5). Salt bridges represented one of the most stable electrostatic interactions at protein–ligand interfaces, critically contributing to the conformational stability of macromolecular complexes [91]. The cation–π interactions between arginine residues and aromatic ligands demonstrated robust stability [92]. Thus, it could be inferred that the *3P* sample exhibited markedly higher values in texture parameters such as hardness, gumminess, and chewiness (Figure 4), which might have resulted from its high level of isoquercitrin. EGCG possessed a structurally complex framework characterized by two aromatic rings, multiple ester groups, and numerous phenolic hydroxyl sites, leading to multiple binding sites for the glutenin–polyphenol–starch complex. The presence of multiple reactive sites on EGCG enabled it to act as a “molecular bridge” within composite networks, linking multiple protein–starch substructures and facilitating spatial aggregation and network reorganization of the macromolecular complex [90,93]. This mechanism may further explain the observed enhancements in hardness and chewiness.

Accordingly, molecular docking suggests spontaneous ternary complex formation between phenolics, glutenin, and starch via diverse interactions (hydrogen bonds, hydrophobic interactions, π–cation interactions, salt bridges). Glutenin–starch binding is primarily hydrogen bond-mediated for dough texture stability, while glutenin–phenolic interactions involve diverse hydrophobic/π/salt bridges, where EGCG and quercetin enhance chewiness through multipoint binding, whereas hydroxybenzoic acid reduces gumminess by disrupting networks. Arg-86 and Arg-649 serve as critical interfacial hubs in ternary complexes, integrating starch hydrogen bonds with phenolic π/electrostatic interactions to govern macromolecular integrity. Nevertheless, it should be emphasized that molecular docking provides computational predictions of potential binding modes between polyphenols and starch/glutenin. Due to the inherent limitations of this in silico technique—such as simplified environmental assumptions—the docking results should be interpreted as hypothesis-generating rather than as experimental proof. Moreover, previous studies have applied various spectroscopic and thermodynamic methods to verify that similar non-covalent interactions indeed occur between polyphenols and macronutrients [88,94]. Therefore, based on the existing literature, the molecular docking predictions in this study are considered experimentally plausible, with key binding residues (e.g., Arg-86 and Arg-649) supported by a reasonable molecular basis, thereby providing a theoretical foundation for subsequent empirical validation.

## 4. Conclusions and Future Perspectives

This study demonstrates that *Rosa roxburghii* pomace particle size (0.1–250 µm) critically governs noodle functionality through polyphenol-mediated mechanisms. The 1–10 µm range emerged as optimal for maximizing the release of total phenolics from the pomace. In boiled noodles, phenolics released from 0.1–1 µm pomace were optimal for maximizing total phenolic retention and those from 60–80 µm pomace were optimal for maximizing the retention of selected phenolic compounds. Fresh noodles exhibited reduced hardness/gumminess (notably in noodles containing phenolics extracted from 1–40 µm pomace). Their boiled counterparts showed increased chewiness/adhesiveness. Cooking properties revealed TPC-driven water absorption reduction and elevated cooking loss due to starch–polyphenol interactions. Boiling induced phenolic degradation/transformation (e.g., gallic acid to hydroxybenzoic acid), though net formation and degradation mechanisms require further study. All phenolic-enriched noodles suppressed starch digestibility and displayed enhanced antioxidant capacity. Quadratic regression models quantitatively linked particle size to key properties, including fresh noodle texture (*R*^2^ = 0.845–0.94), phenolic profiles (*R*^2^ = 0.810–0.938), and boiled noodle antioxidant capacity (*R*^2^ = 0.902), establishing predictive relationships. Although the 0.1–1 μm particle size group showed superior polyphenol release and functionality, such ultrafine grinding requires substantial energy input and poses considerable challenges for industrial-scale implementation, thereby limiting its practical applicability. Future research should explore more feasible strategies such as moderate particle size reduction, enzymatic hydrolysis, or assisted extraction to achieve a balance between functional enhancement and industrial viability.

Variations in both polyphenolic profiles and noodle textural properties are mechanistically attributed to molecular-scale interactions between phenolic compounds (EGCG, hydroxybenzoic acid, gallic acid, quercetin, and isoquercitrin) and noodle structural components (gluten–starch matrix) via diverse interactions (hydrogen bonds, hydrophobic contacts, π–cation stacking, salt bridges). Within these complexes, Arg-86 and Arg-649 may serve as critical interfacial hubs integrating starch hydrogen bonds with phenolic π/electrostatic interactions to govern macromolecular integrity. Spectroscopic and thermodynamic methods could be employed to characterize the structural features of polyphenol–protein and polyphenol–starch complexes, thereby verifying the rationality of the predicted binding sites (e.g., Arg-86, Arg-649) and elucidating their specific influences on dough texture, antioxidant performance, and starch digestibility.

As noodles are ultimately a consumer-oriented food product, sensory attributes such as flavor, mouthfeel, and aroma play a crucial role in overall acceptability. Nevertheless, these perceptual qualities remain difficult to directly associate with molecular interactions. Some studies have reported that tea polyphenols enhanced elasticity, whereas others observed increased bitterness depending on concentration, oxidation level, and matrix composition [56,88,95]. To date, no conclusive evidence has demonstrated that polyphenol–protein or polyphenol–starch interactions directly drive sensory changes. Most molecular-level sensory studies have focused on peptide–taste receptor systems [96,97], while the potential influence of polyphenol–glutenin/starch interactions on sensory attributes such as flavor, mouthfeel, and overall acceptance remains largely uncertain. Therefore, future investigations should integrate sensory evaluation with flavor chemistry to establish a comprehensive structure–function–perception framework for polyphenol-enriched food systems, thereby bridging the current gap between molecular mechanisms and consumer sensory experience.

## Figures and Tables

**Table 1:** Labels and descriptions of noodle samples with phenolic extracts.

Noodle Sample Label		Descriptions
Fresh	Boiled	
0	0	Without phenolic extract addition
1P	1P	Added phenolic extract from pomace (particle size: 0.1–1 μm)
2P	2P	Added phenolic extract from pomace (particle size: 1–10 μm)
3P	3P	Added phenolic extract from pomace (particle size: 20–40 μm)
4P	4P	Added phenolic extract from pomace (particle size: 60–80 μm)
5P	5P	Added phenolic extract from pomace (particle size: 250 μm)

**Table 2:** Total phenolic contents and their retention rate in the noodles with the addition of these phenolic extracts.

Samples	Total Polyphenols Content (mg GAE/g DW)		Retention Rate (%)
	Fresh	Boiled	
1P/1P	6.34 ± 0.15 b	1.06 ± 0.06 a	16.72 ± 1.09 a
2P/2P	7.87 ± 0.16 a	0.67 ± 0.05 bc	8.51 ± 0.45 e
3P/3P	5.80 ± 0.17 c	0.70 ± 0.06 b	12.07 ± 1.29 b
4P/4P	5.53 ± 0.05 d	0.64 ± 0.07 c	11.03 ± 0.76 d
5P/5P	4.77 ± 0.07 e	0.55 ± 0.03 d	11.50 ± 0.40 c

Different letters in the same row donate significant differences (*p* < 0.05). No detectable polyphenols were found in the control noodles (without extract addition).

**Table 3:** The phenolic compound contents (μg/g) of the noodles with the addition of phenolic extract from *Rosa roxburghii* pomace with different particle sizes.

Phenols	Status	Control/ Control	1P/*1P*	2P/*2P*	3P/*3P*	4P/*4P*	5P/*5P*
GA	F	--	5.48 ± 1.52 b	4.19 ± 2.31 bc	4.64 ± 0.60 bc	2.84 ± 0.46 c	9.58 ± 0.67 a
	B	--	8.12 ± 3.73 a	4.47 ± 2.01 b	4.47 ± 0.23 b	6.87 ± 1.24 ab	5.06 ± 0.23 ab
HBA	F	--	48.66 ± 0.35 d	124.79 ± 33.74 b	78.26 ± 6.03 c	259.13 ± 0.75 a	26.35 ± 0.24 d
	B	--	27.35 ± 9.29 b	28.40 ± 5.39 b	38.04 ± 4.24 b	123.54 ± 2.01 a	30.82 ± 7.28 b
EGCG	F	--	23.61 ± 3.59 bc	19.98 ± 1.72 c	27.70 ± 0.77 b	--	44.10 ± 2.22 a
	B	--	--	--	35.75 ± 0.43 a	--	35.19 ± 1.31 a
EC	F	--	211.67 ± 16.00 a	55.85 ± 21.03 c	4.25 ± 2.05 d	57.83 ± 5.82 c	90.04 ± 20.57 b
	B	--	22.56 ± 10.37 b	11.20 ± 8.26 b	40.65 ± 18.82 b	18.29 ± 11.28 b	70.45 ± 20.73 a
IQ	F	--	15.08 ± 0.82 b	189.03 ± 68.10 a	54.58 ± 35.50 b	58.33 ± 12.71 b	44.60 ± 22.59 b
	B	--	40.28 ± 5.73 c	71.73 ± 1.80 b	111.20 ± 10.61 a	102.22 ± 0.98 a	30.80 ± 14.22 c
FA	F	--	255.36 ± 7.25 c	486.82 ± 77.68 b	114.29 ± 9.95 d	80.72 ± 23.82 d	948.01 ± 3.66 a
	B	--	30.98 ± 7.97 b	15.30 ± 3.39 bc	21.63 ± 6.79 bc	161.73 ± 14.46 a	14.22 ± 3.40 c
Rutin	F	--	150.74 ± 30.21 a	40.80 ± 6.76 bc	41.96 ± 3.82 b	13.91 ± 3.05 d	14.96 ± 6.64 cd
	B	--	89.53 ± 8.92 b	17.30 ± 11.33 c	19.62 ± 4.99 c	248.76 ± 38.73 a	15.18 ± 8.02 c
Quercetin	F	--	47.02 ± 0.74 a	38.49 ± 7.22 a	40.59 ± 6.82 a	28.37 ± 7.19 b	22.00 ± 0.90 b
	B	--	22.04 ± 0.38 a	27.23 ± 4.79 a	23.76 ± 4.14 a	23.51 ± 1.56 a	24.28 ± 4.14 a
Sum.	F	--	757.61 ± 49.49 c	959.95 ± 117.78 b	366.24 ± 23.65 e	501.12 ± 40.24 d	1199.64 ± 40.24 a
	B	--	240.85 ± 24.73 c	175.62 ± 5.81 d	295.11 ± 17.63 b	687.17 ± 18.54 a	226.00 ± 22.10 c

Different letters in the same row indicate significant differences (*p* < 0.05). F = fresh; B = boiled. FA = ferulic acid; EC = epicatechin; GA = gallic acid; HBA = hydroxybenzoic acid; IQ = isoquercitrin.

**Table 4:** The color parameters (L*, a*, b*) of the noodles with the addition of phenolic extract from *Rosa roxburghii* pomace with different particle sizes. Values are presented as mean ± standard deviation (*n* = 3). Different letters in the same row donate significant differences (*p* < 0.05).

	Samples	L*	a*	b*	Δ*E*
Fresh	0	81.13 ± 0.49 a	1.73 ± 0.28 g	15.62 ± 0.22 bc	--
	1P	69.95 ± 0.36 d	7.72 ± 0.22 f	29.05 ± 0.67 a	18.48 ± 0.58 c
	2P	70.63 ± 0.30 c	8.47 ± 0.4 e	28.44 ± 0.3 a	17.89 ± 0.26 c
	3P	68.72 ± 0.25 e	9.64 ± 0.71 c	29.62 ± 1.23 a	20.33 ± 0.96 a
	4P	69.81 ± 0.44 d	9.02 ± 0.19 d	29.48 ± 0.85 a	19.32 ± 0.72 b
	5P	69.9 ± 0.61 d	8.15 ± 0.09 e	28.66 ± 0.77 a	18.38 ± 0.49 c
Boiled	0	76.66 ± 0.35 b	0.91 ± 0.19 h	14.05 ± 0.57 c	--
	1P	58.24 ± 0.12 gh	10.68 ± 0.17 a	30.31 ± 0.52 a	26.44 ± 0.34 ab
	2P	57.8 ± 0.55 hi	10.25 ± 0.43 b	30.1 ± 1.49 a	26.48 ± 1.35 ab
	3P	60.45 ± 0.69 f	10.66 ± 0.33 a	29.97 ± 0.93 a	24.73 ± 1.01 ab
	4P	57.59 ± 0.46 i	10.96 ± 0.36 a	31.86 ± 0.81 a	27.96 ± 0.94 a
	5P	58.74 ± 0.61 g	11.01 ± 0.29 a	20.1 ± 12.18 b	23.78 ± 4.06 b

**Table 5:** Molecular docking of phenolic compounds influencing noodle texture.

Receptors	Ligands	Acting Forces	The Binding Sites	Minimum Binding Energy (kal/mol)
Glutenin	Starch, EGCG	Hydrogen bonds	Ser-82 *, Arg-86 *, Arg-77 *, Arg-649 *#, Ser-78 *, Cys-66 *	−8.1
		Hydrophobic interactions	Arg-77 #, Pro-645 #, Val-81 #, Thr-642 #	
	Starch, hydroxybenzoic acid	Hydrogen bonds	Gln-638 *, Thr-642 *, Arg-86, Glu-24 *, Arg-27 *, Lys-75 *, Ser-78 *, Ser-82 *, Arg-77 *#, Gly-652 #	−7.2
		Hydrophobic interactions	Arg-70#	
	Starch, gallic acid	Hydrophobic interactions	Ser-78 *, Gln-627 *, Gln-90 *, Arg-86 *#, Gln-28 *, Ala-629 *, Ser-631 *, Gln-83 #	−7.9
		π–cation interactions	Arg-86 #	
		Salt bridges	Arg-86 #	
	Starch, quercetin	Hydrogen bonds	Leu-35 *, Arg-44 *, Gln-83 *, Gln-90 *, Gln-627 *, Gln-28 *, Arg-86 *, Glu-37 *, Glu-24 #	−8.4
		Hydrophobic interactions	Gln-28 #,Val-79 #	
	Starch, isoquercitrin	Hydrogen bonds	Ser-82 *, Thr-646 *, Glu-24 *, Gln-638 *, Arg-649 #, Arg-77 #	−7.4
		Hydrophobic interactions	Pro-645 #	
		π–cation interactions	Arg-649 #	
		Salt bridges	Arg-649 #	

* Refers to the binding sites between glutenin and starch. # Refers to the binding sites between glutenin and phenolic compounds.

## Data Availability

The data presented in this study are available on request from the corresponding author. The data are not publicly available due to institutional policy and ongoing related research.

## Supplementary Materials

The following supporting information can be downloaded at https://www.mdpi.com/article/10.3390/foods14213679/s1. Table S1: The quadratic fitting equations between polyphenol extracts from *Rosa roxburghii* pomace of different particle sizes (p) and the corresponding properties of uncooked noodles. Table S2: The quadratic fitting equations between polyphenol extracts from *Rosa roxburghii* pomace of different particle sizes (p) and the corresponding properties of boiled noodles.
