# Supplementary material for: Effect of Polyphenols Extracted from Rosa roxburghii Tartt Pomace with Different Particle Sizes on Quality and Biological Activity of Noodles: A View of Molecular Interaction
Source: Foods. 2025 Oct 28;14(21):3679. doi: 10.3390/foods14213679 (PMC12610746; doi:10.3390/foods14213679)
Supplement: Supplementary file 1 [file foods-14-03679-s001.zip › foods-3857408-supplementary.pdf]

Supplementary Table S1 The quadratic equation fitting equations between polyphenol extracts from *Rosa roxburghii* pomace of different particle sizes (p) and uncooked noodles' corresponding properties

	Fresh		
	Quadratic equation	R^2	F
Hardness	y=3146.041+16.382p-0.040p2	0.755	3.078
Adhesiveness	y=-23.449-0.089p+0.001p2	0.730	2.698
Springiness	y=0.356+0.001p-3.316×10-6 p2	0.940	15.609
Cohesiveness	y=0.402 + 1.834×10-4 p -9.376×10-7 p2	0.156	0.185
Gumminess	y=1274.585 + 7.481p -0.021p2	0.740	2.841
Chewiness	y=485.777 + 3.354p -0.009p2	0.845	5.450
Resilience	y=0.173 + 3.830×10-4p -6.206×10-7p2	0.872	6.797
DPPH	y=0.861 -1.355p +1.542×10-7p2	0.426	0.742
Gallic acid	y=5.507-0.049p+2.694×10-4p2	0.951	19.259
Hydroxybenzoic acid	y=67.831+3.801p-0.016p2	0.810	4.256
EGCG	y=21.801+0.074p+5.904×10-5p2	0.981	26.172
Epicatechin	y=134.071-1.688p+0.006p2	0.266	0.362
Iso-quercitrin	y=102.440-0.835p+0.002p2	0.147	0.172
Ferulic acid	y=386.267-7.009p+0.037p2	0.931	13.427
Rutin	y=101.018-1.719p+0.005p2	0.540	1.172
Quercetin	y=44.044-0.287p+0.001p2	0.878	7.210
TPC a	y=865.249-8.280p+0.038p2	0.920	11.565
TPC b	y=6.951-0.033p+9.598×10-5p2	0.701	2.344

[a] TPC refers to the total content of individual phenolic compounds quantified by HPLC (μg/g).
[b] TPC was determined by Folin-Ciocalteu assay (mgGAE/g DW).

Supplementary Table S2 The quadratic equation fitting equations between polyphenol extracts from *Rosa roxburghii* pomace with different particle sizes (p) and boiled noodles' corresponding

	Boiled		
	Quadratic equations	R^2	F
Hardness	y=3971.802-0.944p-0.005p2	0.453	0.827
Adhesiveness	y=256.003-1.123p+0.004p2	0.833	4.971
Springiness	y=0.869-0.001p+3.598×10-6p2	0.556	1.252
Cohesiveness	y=0.590-4.784×10-4p+2.235×10-6p2	0.821	4.51
Gumminess	y=2192.377+2.813p-0.017 p2	0.599	1.491
Chewiness	y=1821.737+1.866p-0.010 p2	0.151	0.178
Resilience	y=0.232+2.969×10-5p-3.166×10-7p2	0.642	1.790
DPPH	y=0.474-0.006p+2.062×10-5p2	0.902	9.231
WAR	y=88.792+0.090p-2.905×10-4p2	0.700	2.336
CLR	y=5.636+0.017p-5.992×10-5p2	0.398	0.661
Gallic acid	y=5.831+0.010p-5.007×10-5p2	0.068	0.074
Hydroxybenzoic acid	y=20.382+2.070p-0.008 p2	0.918	11.151
EGCG	y=8.108+0.066p+ 1.627×10-4p2	0.358	0.558
Epicatechin	y=22.468+0.001p+0.001p2	0.781	3.572
Iso-quercitrin	y=63.616+1.130p-0.005 p2	0.719	2.554
Ferulic acid	y=8.600+2.982p-0.012 p2	0.843	5.356
Rutin	y=25.814+4.165p-0.017 p2	0.699	2.321
Quercetin	y=24.522-0.024p+9.034×10-5p2	0.054	0.058
TPC a	y=179.342+10.400p-0.041 p2	0.938	15.207
TPC b	y=0.848-0.005p+1.431×10-5p2	0.472	0.896

[a] TPC refers to the total content of individual phenolic compounds quantified by HPLC (μg/g).

[b] TPC was determined by Folin-Ciocalteu assay (mgGAE/g DW).
